# A retrospective observational study of characteristics and outcomes of older patients referred to a hospital-based social prescribing programme

**DOI:** 10.3389/fpubh.2026.1762593

**Published:** 2026-02-11

**Authors:** Bernie McGowan, Roman Romero-Ortuno, Louise Brennan, Evan Murphy, Gerard Boyle, David Robinson

**Affiliations:** 1 Mercer’s Institute for Successful Ageing, St James’s Hospital, Dublin, Ireland; 2 Discipline of Clinical Medicine, School of Medicine, Trinity College Dublin, St James’s Hospital, Dublin, Ireland; 3 Discipline of Medical Gerontology, School of Medicine, Trinity College Dublin, Mercer’s Institute for Successful Ageing, St James’s Hospital, Dublin, Ireland; 4 Department of Medical Physics and Bioengineering (MPBE), St James’s Hospital, Dublin, Ireland

**Keywords:** geriatrics, integrated care, social prescribing, wellbeing, WEMWBS, WHO-5

## Abstract

**Introduction:**

Social Prescribing (SP) links people to non-medical supports to address social determinants of health (SDH) and improve wellbeing. To date, there has been little evidence published on the use or outcomes of SP with older adults in a hospital setting. Here, we described patient characteristics, engagement, and pre–post wellbeing outcomes in a hospital-based SP programme for older people.

**Materials and methods:**

Retrospective observational cohort of all patients aged ≥65 referred from a tertiary hospital to a dedicated link-worker SP service (Dublin, Ireland), May 2020–October 2023 (*n* = 370). Wellbeing was assessed with the Warwick-Edinburgh Mental Wellbeing Scale (WEMWBS) and WHO-5 at baseline and end of programme for those with paired data. Descriptive statistics and paired tests were used.

**Results:**

Mean age 80.1 (SD 7.7); 66% female; 65% lived alone; 36% with dementia; mean comorbidities 4.8. Two-hundred ten completed the programme. Among completers with paired measures, WEMWBS increased from 50.2 to 54.8 (*p* = 0.006, *n* = 94) and WHO-5 from 55.5 to 68.7 (*p* = 0.017, *n* = 90).

**Discussion:**

Hospital-based SP for older adults was feasible with measurable improvements in wellbeing among completers. There were barriers to engagement that have wider public health policy implications.

## 1 Introduction

### 1.1 Social determinants of health

Up to 90% of health outcomes are due to the Social Determinants of Health (SDH) which include behaviour, environment and social connectedness (1): these are not addressed in a traditional medical consultation. Social isolation, for instance, is associated with increased risk of cardiovascular disease, poor cognitive function, immunoparesis, and depression (2–4). Failing to address SDH can result in repeated healthcare utilization, at primary and secondary care level.

Healthcare systems are reactive and focused on acute care, largely ignoring SDH. They are increasingly strained in providing for an ageing world. Community services that address SDH tend to operate in a separate silo to healthcare. It has been argued that this fragmented approach should be replaced by a more integrated, holistic model of care encompassing healthy ageing as well as disease, reducing the risk that health systems become overwhelmed (5).

### 1.2 Social prescribing

Social Prescribing (SP) is a means for trusted healthcare professionals to refer a patient to a link worker who then connects the patient to personalized community-based supports for health and wellbeing (6).

Link workers have a profound knowledge of local and community services and can connect patients to groups and activities to increase social engagement, reduce sedentariness and address or improve mental health. Community resources include dance classes, men’s sheds, walking groups, peer led healthy cooking classes, smoking cessation groups and statutory services. Link workers are usually non-clinical and will have more dedicated time than clinical personnel for direct patient engagement. The social prescribing approach addresses the SDH, and connects the silos of health and social care. It aligns closely with principles of geriatric medicine which value non-pharmacological interventions and community engagement.

### 1.3 Social prescribing practice internationally

International models vary, but the growing consensus is that SP requires referral by a healthcare professional to a link worker (6). Link workers have been described as “the people on the bridge”, who connect the silos of health (primary care and secondary care) and community and voluntary sectors.

Evidence for the effectiveness of this approach is still emerging. Several studies of social prescribing show improvement in subjective measures of health and wellbeing (7, 8), with others indicating reduced healthcare utilization, including fewer visits to primary care, or outpatient and emergency department visits (9–15).

Existing social prescribing programmes are broad in design, rarely targeting a particular disease or demographic. This can lead to variability in outcomes (16). However, there are several rigorous randomized controlled trials in development (17, 18).

Most social prescribing projects are community based: secondary care projects are uncommon. Secondary care settings often serve patients with higher levels of acuity, complexity, and multimorbidity. These populations present a challenge: while they may benefit substantially from a health and well-being approach, they also require high levels of medical care. Few hospital-based projects have been rigorously evaluated: most studies were limited to pilots or transitional care interventions (19), however there is emerging evidence that hospital-based social prescribing for paediatric patients with high complexity can reduce unmet need and generate social value (20).

### 1.4 Social prescribing practice in Ireland

Social prescribing began as a grassroots movement in most countries, including Ireland. Irish geriatricians quickly recognized an alignment of social prescribing with the non-pharmacological, holistic ethos of medical gerontology (21).

Stakeholders from the community and voluntary sectors and the health sector in both Ireland and Northern Ireland created the All-Ireland Social Prescribing Network (AISPN). The AISPN partnered with Ireland’s Health Service Executive (HSE) to develop the National Framework for Social Prescribing (22).

This collaboration led to the establishment of an innovative SP service within the geriatric department of St James’s Hospital, located in Dublin’s inner city, an area of considerable social deprivation. The origins and policy context of this service have been described elsewhere (23).

### 1.5 Aims and objectives

The aim of this study was to describe the demographic and clinical characteristics of older adults referred to a hospital-based SP service, the reasons for these referrals and changes in wellbeing scores before and after the intervention.

## 2 Materials and methods

This retrospective observational study included all patients referred to the SP service between May 2020 and October 2023. All referrals originated within the hospital’s Department of Geriatric Medicine. Patients were referred either from outpatient clinics or prior to discharge following an acute hospital admission. Participants were over 65 years of age.

Data collected included demographic details, source and reason for referral, comorbidities, social circumstances, and engagement with the SP service (e.g., number of contacts, types of interventions, and documented outcomes).

### 2.1 Wellbeing outcome measures

Measures of health and wellbeing were obtained using the Warwick–Edinburgh Mental Well-being Scale (WEMWBS, (24)), the WHO-5 Well-being Index (25) and the Measure Yourself Concerns and Wellbeing questionnaire (MYCaW, (26)). These were administered at baseline and repeated at the conclusion of the programme where feasible.

The WEMWBS is a 14-item scale designed to monitor mental wellbeing over time. Scores range from 14 to 70, with higher scores reflecting more positive mental wellbeing. Scores of ≤ 40 indicate probable clinical depression.

The WHO-5 Well-being Index is a five-item questionnaire rated on a 6-point Likert scale that measures recent mental wellbeing. Raw scores from 0 to 25 are multiplied by 4 to generate a percentage. A change of greater than 10% is generally considered clinically significant (25).

The MYCaW allows participants to identify and rate up to 2 of their most pressing concerns using a seven-point scale and to rate overall well-being. In contrast to other standardised tools, it records patient-prioritised outcomes. Higher scores are indicative of a more negative impact of concerns on wellbeing (26).

### 2.2 Statistical analyses

Statistical analyses were conducted using IBM SPSS Statistics version 27 (IBM Corp., Armonk, NY, USA). Descriptive statistics were used to summarise participant characteristics. Categorical variables were described in terms of count and percentage, while continuous variables were presented as mean and standard deviation (SD), or median with interquartile range (IQR), as appropriate.

For participants who completed pre and post evaluation, a General Linear Model (GLM) Repeated Measures analysis was conducted. This analysis controlled for covariates including age, sex, living alone (yes or no), presence of dementia or mild cognitive impairment (MCI), number of comorbidities, and duration of participation in the SP programme. The GLM Repeated Measures analysis focused on modeling the estimated marginal means of key outcome measures, namely WEMWBS, WHO-5 Well-Being Index, and MYCaW Scales.

### 2.3 Ethical approval

As a retrospective observational study of routinely collected data as part of service evaluation, formal consent was not sought from participants. Ethical approval for the study was obtained from St James’s Hospital/Tallaght University Hospital Research Ethics Committee Secretariat (JREC Reference: 2020–06 List 23).

Patients and members of the public were not involved in the design, conduct, reporting, or dissemination plans of this study, as it was a retrospective service evaluation based on routinely collected data.

## 3 Results

### 3.1 Demographic, social and health profile of participants referred

Three hundred seventy individuals aged 65 and over were referred to the SP programme over the study duration. Characteristics of patients referred are presented in Table 1.

**Table 1:** Characteristics of older patients referred (*n* = 370).

Characteristic	*N*	Percentage
Gender		
Female	243	65.7%
Male	127	34.3%
Age group		
≤75 years	108	29.2%
75–85 years	167	45.1%
> 85 years	93	25.1%
Not documented	2	0.5%
Living with		
Alone	240	64.9%
Partner	84	22.7%
Daughter/son/niece	33	8.9%
Sibling	4	1.1%
Other	6	1.6%
Not documented	3	0.8%
Reason for referral		
Social Isolation and Loneliness	197	53.2%
Dementia-specific cognitive activities	96	25.9%
Re-connection following period of illness	44	11.9%
Anxiety/panic attacks/low mood	17	4.6%
Caregiver stress	8	2.2%
Other	5	1.4%
Not documented	3	0.8%
Cognition		
Living with dementia	132	35.7%
Mild cognitive impairment	74	20.0%
Normal cognition	164	44.3%

Mean age of patients referred was 80.1 (SD 7.7), 66% were female, and 65% lived alone.

Less than half had normal cognition: 36% were living with a diagnosis of dementia, and a further 20% had a diagnosis of MCI. The mean number of comorbidities was 4.8 (SD 2.5) (data available for *n* = 360).

Social isolation and loneliness were the commonest reasons for referral, followed by people living with dementia who were referred for dementia-specific activities to increase social or cognitive stimulation. An additional 12% (*n* = 44) were referred for re-engagement in community life following a period of functional decline or illness.

### 3.2 Service delivery

Mean time from referral to first SP contact was 88.1 days (SD 60.0). Of the 370 patients, the first point of contact occurred on the ward prior to discharge for 196 patients (53.0%), in their own homes for 152 (41.1%), and in outpatient clinics for 13 (3.5%).

The mean number of contacts per patient was 5.8 (SD 5.0), and the mean length of time on the programme was 102.6 days (SD 112.0), with a median 62 days (IQR 125) (Table 2).

**Table 2:** Service delivery characteristics.

Characteristic	*n*	Mean (SD)/*n* (%)
Time from referral to first SP contact (days)	365	88.1 (60.0)
First point of contact	370	
Ward prior to discharge	196	53.0%
Own home	152	41.1%
Outpatient clinic	13	3.5%
Data not available	9	2.4%
Number of contacts per patient	369	5.8 (5.0)
Time on programme (days)	346	102.6 (112.0)

### 3.3 Outcomes following referral

**Figure 1:**
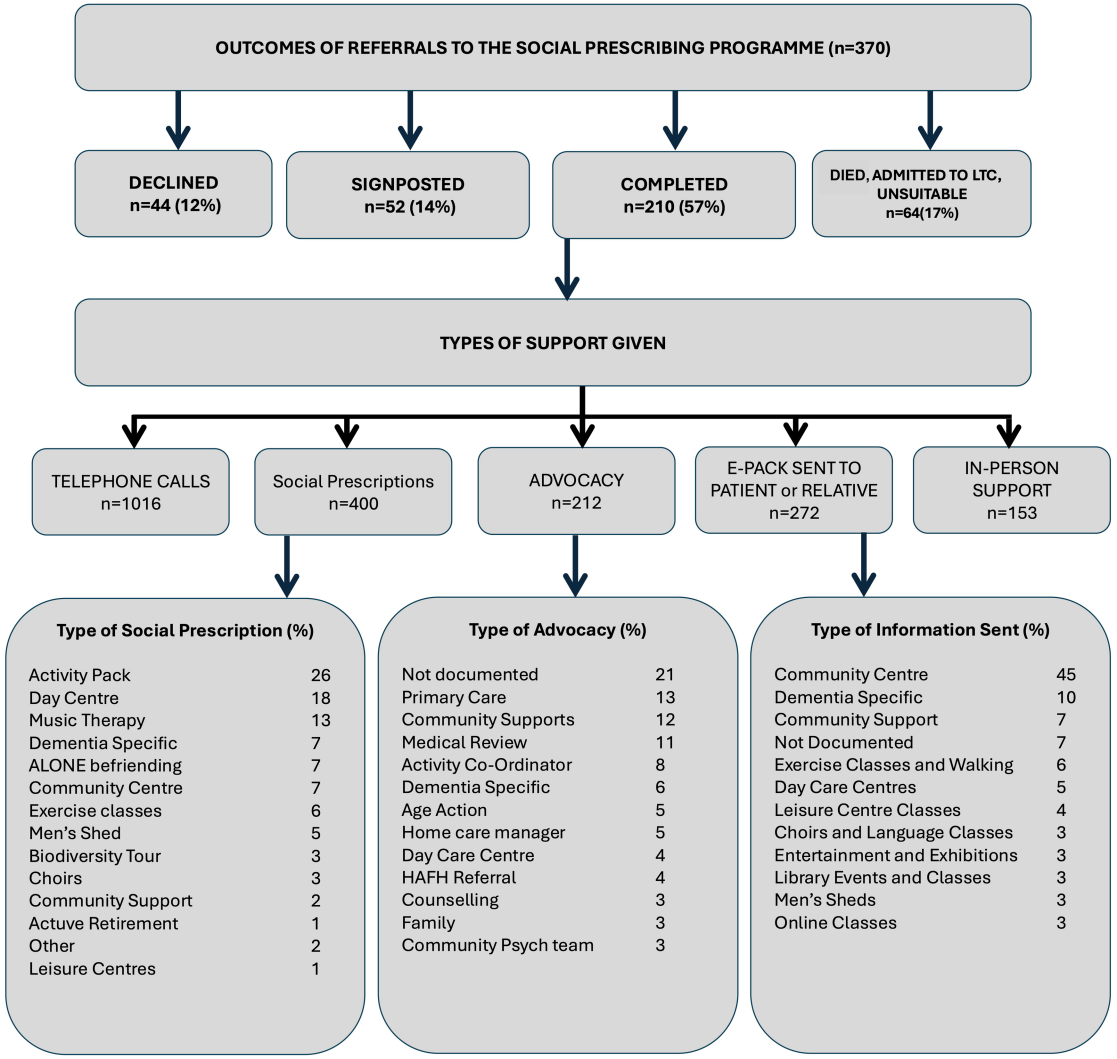
Outcomes of referrals to the Social Prescribing Programme (*n* = 370). Proportions of patients who declined, were signposted, completed the programme, or had other outcomes (died, admission to long-term care [LTC], or unsuitability). Types of support included telephone calls, social prescriptions, advocacy, e-packs, and in-person support. LTC, long-term care; HAFH, Healthy Age Friendly Homes.

Of 370 patients referred, slightly over half completed the SP intervention, defined as engagement with the link worker with co-prescription of an intervention (56.8%, *n* = 210). Sixty-four patients (17.3%) were unable to engage due to deteriorating health, hospital admission, admission to long-term care, or living outside catchment. A further 52 patients (14.1%) were signposted only, i.e., provided with information or referrals but without formal enrolment. About one in eight (11.9%, *n* = 44) declined the service when contacted, citing sufficient community engagement or family rather than personal preference for engagement (Figure 1).

### 3.4 Types of support given

For the 210 patients who completed the SP programme, a total of 2,053 contacts were recorded, with a mean of 10 contacts per patient. These contacts were of four types: (1) SP telephone support, (2) advocacy, (3) referral to appropriate community services, and (4) information emailed or posted to patients or a family member in relation to community and social activities in their area (Figure 1).

These categories represented different modes of link-worker activity—social prescriptions (referrals to community activities), advocacy (active liaison or problem-solving with services), and information provision (one-way sharing of resources)—and were not mutually exclusive: the same thematic area (e.g., dementia-specific support) could involve more than one mode of activity.

Each patient received a mean of 2 social prescriptions: 26% of prescriptions were for Online Activity Packs, a further 23% were for Community Centre and Family Resource Centre activities such as exercise classes, art, knitting and crochet classes, men’s sheds, choirs and walking groups. Eighteen percent of prescriptions were for Day Care Centres. Befriending services and biodiversity tours were also among the social prescriptions. Participants who completed the programme received a mean of 5 telephone calls. In total, 38% of advocacy work involved liaising with Primary Care Teams such as public health nurses, general practitioners (GPs) and social workers to address medical and social issues, liaising with managers of home care packages (HCPs), dementia specific supports and services, community psychiatric teams or hospital-based medical personnel.

### 3.5 Comparison of mental health and well-being scores pre- and post- participation

**Figure 2:**
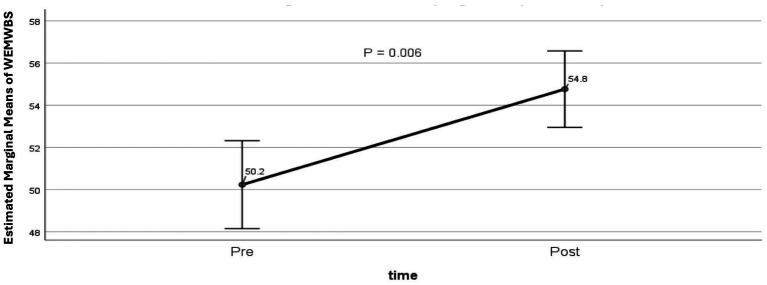
Warwick-Edinburgh Mental Wellbeing Scale (WEMWBS) scores at baseline and post- completion of programme. Estimated marginal means of WEMWBS at baseline and completion, controlling for age, sex, living alone, dementia or mild cognitive impairment, number of comorbidities, and length of time on the SP programme: 50.2 vs. 54.8, *p* = <0.01 (GLM repeated measures, *n* = 94).

Among participants who completed both pre- and post-evaluations using the WEMWBS (*n* = 94), there was a statistically significant improvement in mental wellbeing, with adjusted mean scores increasing from 50.2 at baseline to 54.8 post-intervention (*p* = 0.006; Figure 2). The analysis controlled for age, sex, living alone, presence of dementia or MCI, number of comorbidities, and duration in the SP programme. Gains were demonstrated in wellbeing domains including functioning, social relationships, sense of purpose, and overall happiness.

**Figure 3:**
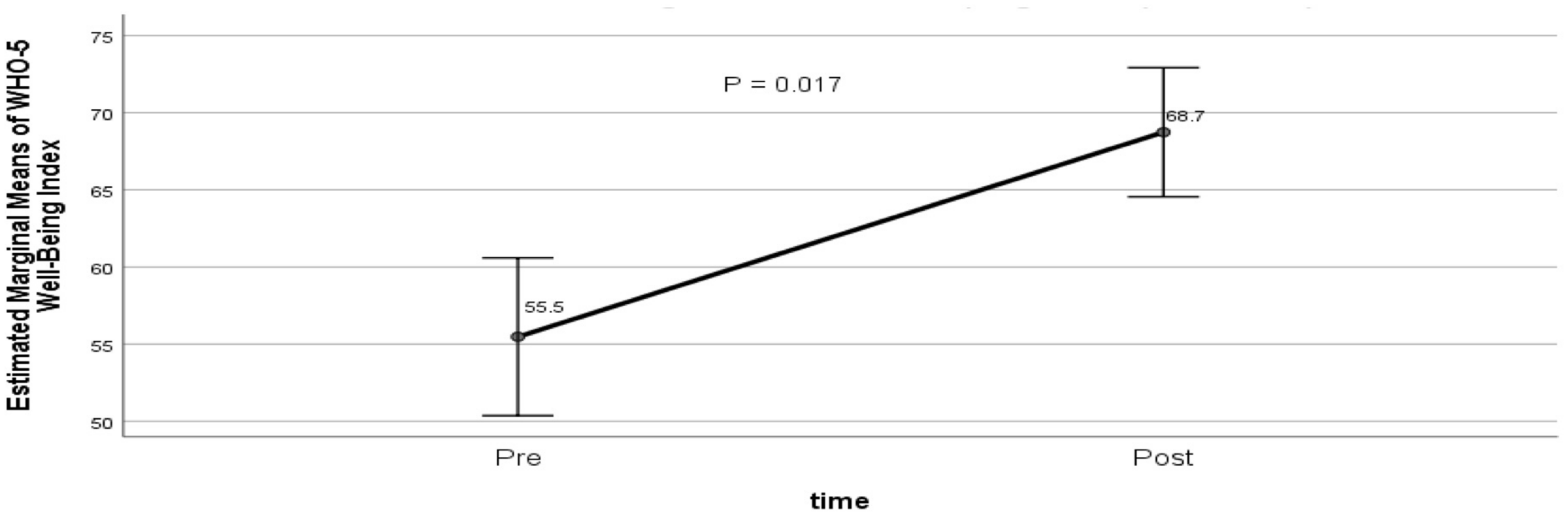
WHO-5 well-being index before and after the social prescribing programme. Estimated marginal means WHO-5 percentage scores (0–100) pre- and post-intervention, adjusted for age, sex, living alone, dementia or mild cognitive impairment, number of comorbidities, and duration of programme participation: 55.5 vs. 68.7, *p* < 0.05 (GLM repeated measures, *n* = 90).

Among participants who completed both pre- and post-evaluations using the WHO-5 Well-Being Index (*n* = 90), there was a statistically significant improvement in wellbeing, with adjusted mean scores increasing from 55.5 at baseline to 68.7 post-intervention (*p* = 0.017; Figure 3).

**Figure 4:**
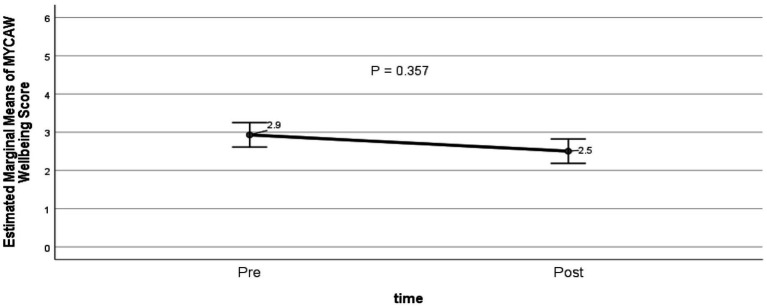
Change in MYCaW wellbeing score at baseline and post-completion of programme. Estimated marginal means adjusted for age, sex, living alone, dementia or mild cognitive impairment, number of comorbidities, and duration of participation in the social prescribing programme. Lower scores indicate better wellbeing (GLM repeated measures, *p* = 0.357; *n* = 88).

Among participants who completed both pre- and post-evaluations using the MYCaW tool (*n* = 88), mean wellbeing scores decreased from 2.9 at baseline to 2.5 post-intervention, indicating an improvement in perceived wellbeing (as a lower MyCaW score reflects better outcomes). However, the improvement in scores did not reach statistical significance (*p* = 0.357; Figure 4).

**Figure 5:**
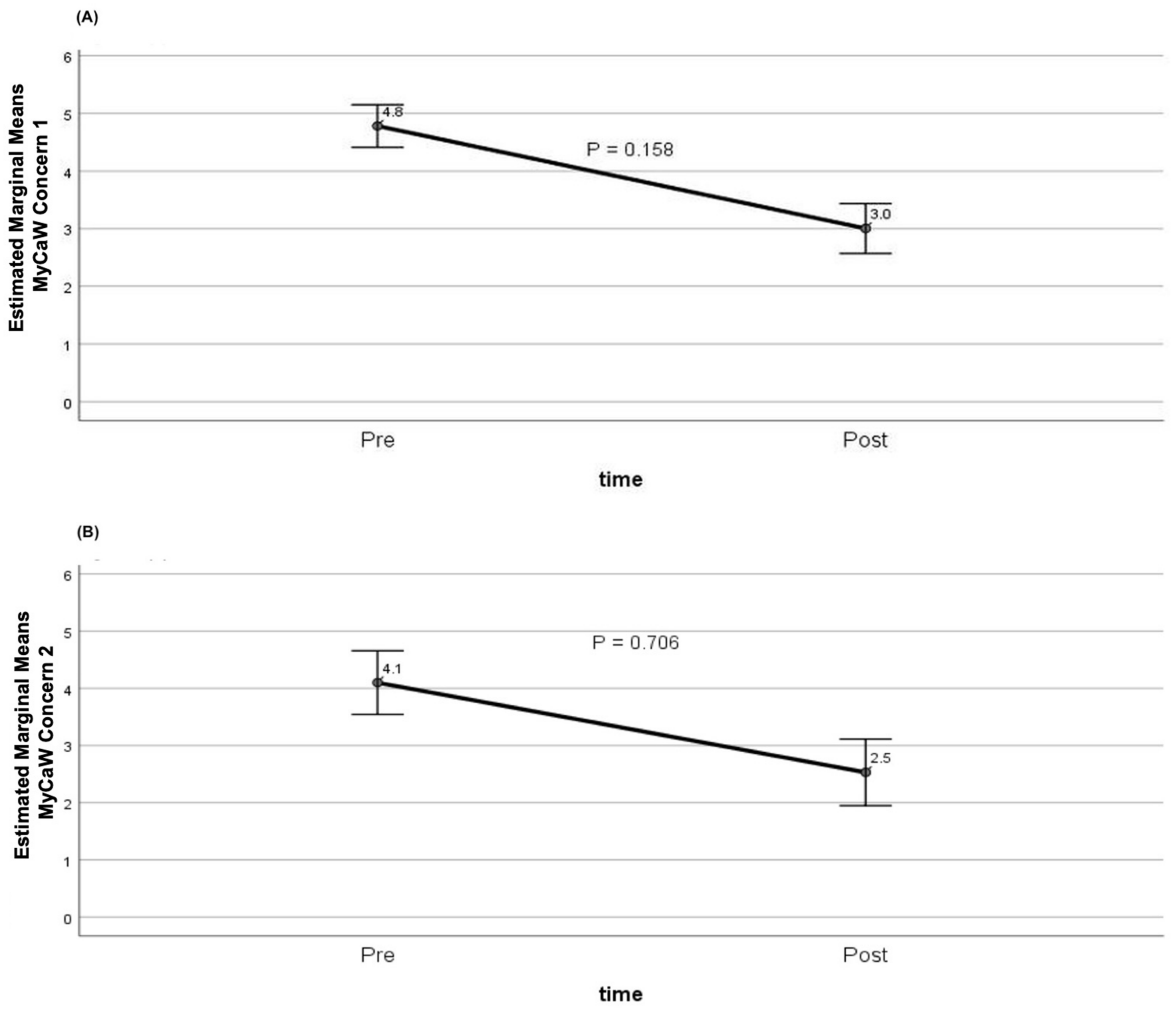
Change in MYCaW concern scores at baseline and post-completion of programme. **(A)** Concern/Problem 1 (*n* = 75); **(B)** Concern/Problem 2 (*n* = 44). Estimated marginal mean MYCaW concern scores adjusted for age, sex, living alone, dementia or mild cognitive impairment, number of comorbidities, and duration of participation in the social prescribing programme. Scores decreased from 4.8 to 3.0 for Concern/Problem 1 and from 4.1 to 2.5 for Concern/Problem 2, indicating reduced impact of concerns on wellbeing; however, these changes did not reach statistical significance (GLM repeated measures, *p* = 0.158 and *p* = 0.706, respectively).

The extent to which participants’ primary and secondary concerns impacted their lives are shown in Figures 5A,B, respectively. For Concern/Problem 1 (*n* = 75), mean scores decreased from 4.8 at baseline to 3.0 post-intervention, while for Concern/Problem 2 (*n* = 44), scores decreased from 4.1 to 2.5. In both cases, lower scores indicate less impact and thus an improvement in wellbeing. However, these improvements did not reach statistical significance (*p* = 0.158 and *p* = 0.706, respectively).

## 4 Discussion

This retrospective evaluation explored the implementation of a SP link worker service for older adults (≥65 years) with multiple long-term conditions in a secondary care setting.

To our knowledge, it is the first study to report on such a service within an Irish acute hospital context.

In contrast to community-based SP projects, participants in this hospital-based cohort had higher levels of medical morbidity and cognitive impairment, with a greater prevalence of dementia, social isolation (living alone), and reliance on formal carer support.

### 4.1 Social isolation and loneliness

Social isolation and loneliness were the main reasons for referral, accounting just over half of all referrals. Loneliness among older adults has been recognized as a significant public health problem over the past decade (27), with the majority of referrals to SP services in Ireland documenting social isolation and loneliness as the most frequent reason given for referral to programs nationally (28).

There is a strong correlation between social isolation and loneliness and adverse health outcomes such as depression (29), decline in cognitive function (30–32), increased frailty (33), cardiovascular disease (34), and increased risk of mortality (35).

Interventions to combat loneliness have not been as effective as interventions for other social and behavioural outcomes (36). Digital interventions are no better (37). There are small to medium effects from psychotherapy (38). Yet, our evaluation demonstrated statistically and clinically significant improvements in two subjective measures of health and well-being: the WEMWBS and WHO-5 Well-being evaluations. Wellbeing scores in the MYCaW improved without showing statistical significance. Our study did not directly measure loneliness – future studies could incorporate validated loneliness scales to determine if improvements in well-being are mediated through changes in loneliness.

### 4.2 Clinically meaningful change

For the 14-item WEMWBS, we estimated a mean improvement of 5 points and previous studies indicate that a change between 3 and 8 points can be considered important. The literature suggests a change of 8 or more points is statistically significant, representing a higher threshold based on 2.77 standard error of measurement (SEM). However, a change of 3 or more points (1 SEM) is still meaningful, as it exceeds the measurement error in most studies (39).

For the WHO-5 Well-being Index, percentage scores are typically used to monitor change. In our evaluation, mean scores increased by 13.2 points (from 55.5 to 68.7), representing a 23.8% improvement, which exceeds the accepted threshold of 10% for clinical significance (25).

### 4.3 Barriers to engagement or completion

Barriers included access to transport, poor mobility, burden of appointments, impact of dementia, and COVID-19 related social anxiety, consistent with community-based social prescribing studies in older adults (40). In addition, hospital-specific barriers reflected a higher level of medical acuity, with substantial loss to follow-up due to clinical deterioration, death, or transition to long-term care—factors less commonly encountered in community-based social prescribing programmes.

The success of a social prescribing programme for older people depends on several factors, with evidence consistently highlighting the central role of the link worker in mitigating barriers to engagement (41).

A majority of older people with chronic conditions are likely to benefit from holistic social prescribing irrespective of age, gender and levels of activation and frailty (11).

A social prescribing project tailored for older people needs to be age-attuned, i.e., holistic, individually tailored and flexible. Our study was conducted in a recognised centre of excellence for care for older persons. The link worker had a past background in nursing so was comfortable interfacing with clinical services.

The study took place during the COVID-19 pandemic with fluctuating restrictions, and availability of health and well-being programmes. This impacted the number of referrals possible. Many activities were digital or home based. Barriers to digital participation included lack of equipment, connection, or digital literacy. It was not possible to examine any impact on hospital or outpatient attendance. Without a control group it is not possible to infer causation.

### 4.4 Strengths and limitations

Study strengths include a large and well-characterized cohort, with pre- and post-intervention measures available. The study provides a contrasting population to healthier community-based cohorts, with higher rates of physical or cognitive impairment, social isolation or reliance on formal care.

Limitations include an ethnically homogenous group, based at one centre. Findings may not be generalisable to other healthcare systems or populations. Higher levels of morbidity may have impacted the intervention, particularly in a cohort of older patients (13).

There was significant loss to follow-up due to morbidity. This may have biased results.

### 4.5 Implications for public health policy

Existing policy frameworks for social prescribing are designed predominately around community programs. This study demonstrates that embedding a link worker within secondary geriatric services in a hospital setting is feasible and can deliver measureable improvements in well-being in a cohort of older adults with frailty or dementia.

The pathways described here could serve as an operational model for commissioning or scaling. Barriers identified are not specific to social prescribing but rather reflect the needs of an ageing population. This highlights structural issues that will require intervention at policy rather than individual level.

## 5 Conclusion

Social prescribing is growing in popularity, driven by policies addressing public health challenges such as an ageing population, rising numbers of long-term conditions, a mental health crisis, and the impact of SDH (42).

Our evaluation demonstrated that it is feasible to set up and maintain a social prescribing pathway in a medically complex population in an acute hospital. The study demonstrated meaningful outcomes in improvements in subjective measures of well-being. The findings from this study can inform the design of other social prescribing projects in secondary care settings.

## Data Availability

The data analyzed in this study is subject to the following licenses/restrictions: data are anonymised and held in secure servers at St James’s Hospital within the jurisdiction of the European Union. Requests to access these datasets should be directed to drobinson@stjames.ie.
